# Elevated Troponins after COVID-19 Hospitalization and Long-Term COVID-19 Symptoms: Incidence, Prognosis, and Clinical Outcomes—Results from a Multi-Center International Prospective Registry (HOPE-2)

**DOI:** 10.3390/jcm13092596

**Published:** 2024-04-28

**Authors:** Ravi Vazirani, Gisela Feltes, Rafael Sánchez-del Hoyo, María C. Viana-Llamas, Sergio Raposeiras-Roubín, Rodolfo Romero, Emilio Alfonso-Rodríguez, Aitor Uribarri, Francesco Santoro, Víctor Becerra-Muñoz, Martino Pepe, Alex F. Castro-Mejía, Jaime Signes-Costa, Adelina Gonzalez, Francisco Marín, Javier Lopez-País, Enrico Cerrato, Olalla Vázquez-Cancela, Carolina Espejo-Paeres, Álvaro López Masjuan, Lazar Velicki, Ibrahim El-Battrawy, Harish Ramakrishna, Antonio Fernandez-Ortiz, Ivan J. Nuñez-Gil

**Affiliations:** 1 Cardiology Department, Hospital Clínico San Carlos, 28040 Madrid, Spain; ravi_94@hotmail.es (R.V.); antonio.fernandezortiz@salud.madrid.org (A.F.-O.); 2 Cardiology Department, Hospital Vithas Arturo Soria, 28043 Madrid, Spain; feltesgisela@yahoo.es; 3 Research Methodological Support Unit and Preventive Department, Hospital Clínico San Carlos, IdISSC, 28040 Madrid, Spain; rafaelsanchezdelhoyo@gmail.com; 4 Cardiology Department, Hospital Universitario de Guadalajara, 19002 Guadalajara, Spain; mdviana@sescam.jccm.es; 5 Cardiology Department, Hospital Universitario Álvaro Cunqueiro, 36312 Vigo, Spain; raposeiras26@hotmail.com; 6 Emergency Department, Hospital Isabel Zendal, Hospital Universitario de Getafe, 28905 Madrid, Spain; rodolfo.romero@salud.madrid.org; 7 Faculty of Biomedical and Health Sciences, Universidad Europea de Madrid, 28670 Villaviciosa de Odón, Spain; 8 Cardiology Department, Bellvitge Biomedical Research Institute (IDIBELL), Hospital Universitario de Bellvitge, 08908 Barcelona, Spain; milloal82@gmail.com; 9 Cardiology Department, Hospital Universitari Vall d’Hebron, 08035 Barcelona, Spain; 10 Cardiology Department, Ospedali Riuniti, 71122 Foggia, Italy; dr.francesco.santoro.it@gmail.com; 11 Cardiology Department, Hospital Clínico Universitario Virgen de la Victoria, 29010 Malaga, Spain; vmbecerram@gmail.com; 12 Division of Cardiology, Department of Interdisciplinary Medicine (D.I.M.), University of Bari Aldo Moro, 70121 Bari, Italy; drmartinopepe@gmail.com; 13 Hospital General del Norte de Guayaquil IESS “Los Ceibos”, Guayaquil 090615, Ecuador; alexfercastromejia@gmail.com; 14 Pneumology Department, Hospital Clínico de Valencia, INCLIVA, University of Valencia, 46010 Valencia, Spain; jaimesignescosta@gmail.com; 15 Anesthesiology Department, Hospital Universitario Infanta Sofia, 28702 Madrid, Spain; adelina.gonzalez.m@gmail.com; 16 Cardiology Department, Hospital Clínico Universitario Virgen de la Arrixaca, IMIB-Arrixaca, CIBERCV, 30120 Murcia, Spain; fcomarino@hotmail.com; 17 Cardiology Department, Complejo Hospitalario Universitario de Ourense, 32004 Orense, Spain; javierlopezpais@hotmail.com; 18 Cardiology Department, San Luigi Gonzaga University Hospital, Orbassano and Rivoli Infermi Hospital, 10098 Rivoli, Italy; enrico.cerrato@gmail.com; 19 Preventive Department, Hospital Universitario de Santiago de Compostela, 15706 Santiago de Compostela, Spain; olalla.vazquez.cancela@sergas.es; 20 Cardiology and Emergency Department, Hospital Universitario Príncipe de Asturias, 28805 Madrid, Spain; carolina.espejo.paeres@gmail.com; 21 Cardiology Department, Hospital Universitario Juan Ramón Jimenez, 21005 Huelva, Spain; alvarolmr93@gmail.com; 22 Faculty of Medicine, University of Novi Sad, 21000 Novi Sad, Serbia; lazar.velicki@mf.uns.ac.rs; 23 Institute of Cardiovascular Diseases Vojvodina, 21204 Sremska Kamenica, Serbia; 24 Institute of Physiology, Department of Cellular and Translational Physiology, Medical Faculty, Ruhr University of Bochum, 44801 Bochum, Germany; ibrahim.elbattrawy2006@gmail.com; 25 Institut für Forschung und Lehre (IFL),Molecular and Experimental Cardiology, Ruhr University of Bochum, 44801 Bochum, Germany; 26 Department of Cardiology, St. Josef-Hospital of the Ruhr University Bochum, 44801 Bochum, Germany; 27 Anesthesiology Department, Mayo Clinic, Rochester, MN 55905, USA; ramakrishna.harish@mayo.edu

**Keywords:** COVID-19, troponin, cardiomyopathy, long-term COVID-19 symptoms, mortality

## Abstract

**Background**: Acute cardiac injury (ACI) after COVID-19 has been linked with unfavorable clinical outcomes, but data on the clinical impact of elevated cardiac troponin on discharge during follow-up are scarce. Our objective is to elucidate the clinical outcome of patients with elevated troponin on discharge after surviving a COVID-19 hospitalization. **Methods**: We conducted an analysis in the prospective registry HOPE-2 (NCT04778020). Only patients discharged alive were selected for analysis, and all-cause death on follow-up was considered as the primary endpoint. As a secondary endpoint, we established any long-term COVID-19 symptoms. HOPE-2 stopped enrolling patients on 31 December 2021, with 9299 patients hospitalized with COVID-19, of which 1805 were deceased during the acute phase. Finally, 2382 patients alive on discharge underwent propensity score matching by relevant baseline variables in a 1:3 fashion, from 56 centers in 8 countries. **Results**: Patients with elevated troponin experienced significantly higher all-cause death during follow-up (log-rank = 27.23, *p* < 0.001), and had a higher chance of experiencing long-term COVID-19 cardiovascular symptoms. Specifically, fatigue and dyspnea (57.7% and 62.8%, with *p*-values of 0.009 and <0.001, respectively) are among the most common. **Conclusions:** After surviving the acute phase, patients with elevated troponin on discharge present increased mortality and long-term COVID-19 symptoms over time, which is clinically relevant in follow-up visits.

## 1. Introduction

The World Health Organization (WHO) officially declared COVID-19 a pandemic on 11 March 2020, which has led to millions of deaths thus far [1].

Acute cardiac injury (ACI) represents a significant complication of COVID-19 [2,3], correlating with increased susceptibility to disease severity [4] and mortality [5]. Cardiac muscle damage can either be inflicted by direct heart damage by the severe acute respiratory syndrome coronavirus 2 (SARS-CoV-2) [6] or by a concurrent condition, such as respiratory failure, sepsis, and systemic inflammation, among others [7,8,9].

Persistent cardiac injury is common among COVID-19 survivors and has been shown to recover in only 44.9% of the patients [2]. It might harbor persistent inflammation with myocarditis-like injury [10] and ventricular impairment [11] detected by cardiac magnetic resonance (CMR) and, even though it might not be a frequent finding, it may represent a clinically relevant topic in COVID-19 survivors. However, the clinical impact of these persistently elevated troponin levels on cardiovascular outcomes and long-term prognosis has yet to be determined [12].

Long-term COVID-19 symptoms can be broadly defined as signs, symptoms, and sequelae that continue or develop after an acute COVID-19 infection for any period of time, with the possibility of severe and life-threatening events even months or years after the infection [13]. It is clinically challenging to establish the cessation of the COVID-19 infection, and prospective data regarding the duration of long-term viral persistence are scarce. Moreover, long-term COVID-19 phenotypes might grow over time, generating confusion among stakeholders and precluding the successful research of effective interventions [13].

Our aim is to study the clinical outcomes and prognosis of COVID-19 survivors who are discharged with elevated troponin levels, as well as its relationship with long-term COVID-19 symptoms in an international prospective multicenter registry.

## 2. Material and Methods

### 2.1. Study Design and Participation Criteria

The registry HOPE-2 (Health Outcome Predictive Evaluation for COVID-19—2, NCT04778020) is an international, investigator-led, prospective study framed with a practical “all comers” approach, not offering financial incentives to researchers. Data supporting this research can be requested from the lead author. Inclusion criteria encompass patients discharged after any in-hospital stay, with a subsequent diagnosis of COVID-19, irrespective of survival status. Diagnostic confirmation was done by throat swabs, and analyzed using real-time reverse transcriptase-polymerase chain reaction assays, following the WHO guidelines. Diagnostic confirmation was waived in case of death before testing in a highly suspected COVID-19 case. Clinical decisions and procedures adhered to by the attending medical team were in line with the established local standards and practices [14].

The HOPE-2 registry received ethical approval from Hospital Clínico San Carlos’ ethics committee (21/128-E), gaining endorsement from local institutional boards or ethics committees. The requirement for written informed consent was not applied given the study’s anonymized and observational nature. Electronic collection of the data was performed using an online database [15]. This manuscript reflects data analysis up to 31 December 2021. Data integrity and draft revision are warranted by the lead researchers at each local facility. A detailed participant list and study definitions have already been published elsewhere [14]. The study design and implementation did not involve input from patients.

### 2.2. Data Acquisition and Study Definitions

The inclusion criteria encompassed patients after any in-hospital admission, from any medical facility, given that they had either a confirmed or a highly suspected diagnosis of COVID-19 (especially if death occurred prior to testing).

Patients were considered to have persistent cardiac troponin elevation if, at the time of discharge, they had a serum troponin (T or I) level above the 99th percentile upper reference limit for the cut-off value used in each hospital.

A pragmatic definition of heart disease was adopted and divided into various groups: arrhythmic, coronary, heart failure or cardiomyopathy, heart valve disease, combined and non-specified, or other heart disease different from the aforementioned (e.g., congenital heart disease) according to the local physicians. Any heart disease was considered when it was stated in the clinical history and/or the patient was receiving medication for such purpose [15].

**Figure 1:**
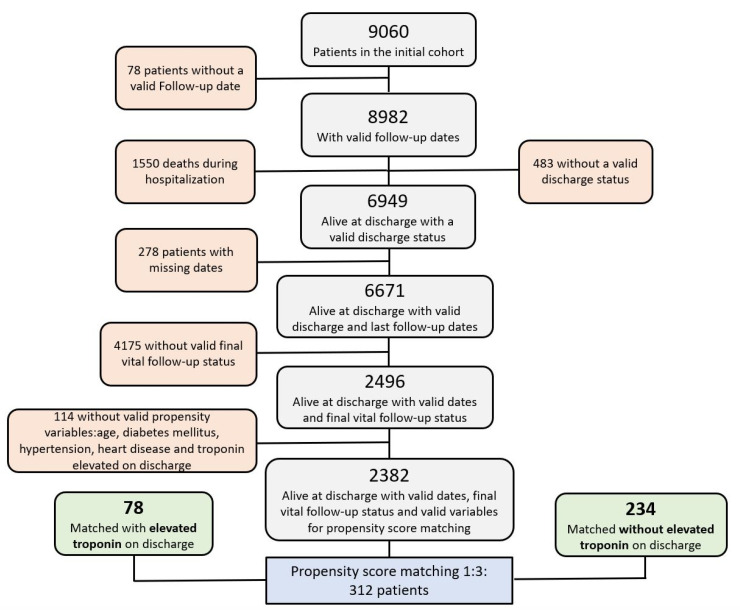
Flowchart describing the stepwise patient selection process, only patients with the needed available variables for analysis were selected for propensity score matching. A total of 4175 patients did not have a structured long-term follow-up, because they came from HOPE-1, a cross-sectional study.

Events occurring during hospitalization (respiratory insufficiency, congestive heart failure, relevant bleeding episodes, upper respiratory tract infection, embolic events, pneumonia, hemoptysis, systemic inflammatory response syndrome, and kidney dysfunction) and during clinical follow-up were assigned by the local physicians’ criteria according to the preestablished definitions. The complete list of variables used in this investigation and their definitions recorded in HOPE-2 have already been published [14]. The flowchart with the detailed patient selection algorithm is shown in Figure 1.

### 2.3. Study Follow-Up and Outcomes

Eligible patients for this research had to be discharged alive and have elevated troponin levels on discharge. All-cause death was the main endpoint. Long-term COVID-19 symptoms, as well as hospital readmissions during follow-up, were considered as secondary endpoints. A structured protocol for long-term COVID-19 symptoms was elaborated according to previous reports [16]. Patient follow-up was carried out either by phone calls with the patient or their family, interviews with their referring physicians, or health records revision [14].

### 2.4. Statistical Analysis

Continuous variables are described as mean and standard deviation, or mean and interquartile range, when appropriate. Categorical variables were reported as frequency (%). Exclusion of missing data (in a listwise fashion) was performed without employing imputation techniques.

A Student *t*-test or Mann–Whitney U test were used to compare continuous variables when needed. A Chi-square test or Fisher’s exact test (when needed) were used to compare categorical variables.

A propensity score for having elevated troponin levels on discharge was calculated from the overall sample based on baseline variables deemed as clinically relevant for the outcome that exhibited a *p*-value ≤0.10 in the univariant analysis: age, diabetes mellitus hypertension, and previous heart disease (Supplementary Table S1).

The propensity score technique was employed to address imbalances in our study groups and mitigate bias arising from the disparity in group sizes.

A 1:3 propensity-matched sample was selected of patients who were discharged alive and had elevated troponin levels on discharge [17]. After the propensity, two balanced groups were produced, thereby allowing for comparisons and eliminating biases that might arise from differences in baseline characteristics.

The nearest neighbor matching method was used for propensity matching. Survival was plotted on Kaplan–Meier curves and assessed with a log-rank test. Survival time was calculated from discharge to the last follow-up date (or death). Relative risk with 95% CI was calculated.

The analysis was performed in the overall selected population as well (Supplementary Tables S2 and S3; Supplementary Figure S1).

Statistical analysis was performed with IBM SPSS statistics v26.0 (SPSS, Inc., Chicago, IL, USA), and R statistical software v. 4.3.1 in the aforementioned analyses. The tests were two-sided, and a *p*-value < 0.05 was considered statistically significant [14].

## 3. Results

Baseline clinical data from the general population discharged alive from the hospital after propensity score matching are described in Table 1. Long-term COVID-19 symptoms incidence in the matched population is described in Table 2.

Patients with elevated troponin levels on discharge were more commonly active smokers, with previous heart disease and had a more abrupt clinical course during hospitalization, with higher rates of heart failure and sepsis (*p* < 0.001). After discharge, they were more likely to be readmitted and showed higher all-cause death rates (*p* < 0.001).

**Figure 2:**
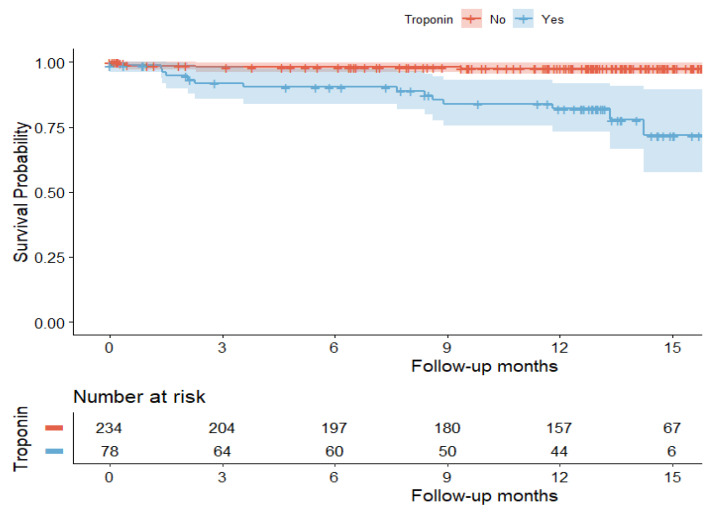
Survival analysis represented by Kaplan–Meier curves (upper part) and number of patients at risk after the end of each time period (lower part). The shaded area surrounding each curve represents the 95% confidence interval.

Survival analysis is presented by Kaplan–Meier curves in Figure 2, demonstrating greater all-cause mortality with a log-rank (Mantel–Cox) test of 27.23 (*p* < 0.001) in patients with elevated troponin after hospital discharge.

Long-term COVID-19 cardiovascular traits were more common in patients with elevated cardiac troponin on discharge, who had higher rates of fatigue, dyspnea, dizziness, palpitations, chest pain, acute coronary syndrome, new onset ventricular dysfunction, and arrhythmia. Even though the number of certain events was reduced, a trend towards increased resting heart rate, atrial fibrillation, pericarditis and/or myocarditis, and inferior limb edema in patients with elevated troponin was observed. No differences were observed in syncope, new onset hypertension, and relevant bleeding during follow-up.

Certain neurological manifestations of long-term COVID-19 were more common in patients with elevated cardiac troponin levels on discharge, such as depression. However, the rest of the studied variables did not exhibit statistically significant differences between groups.

Regarding multisystemic manifestations of long-term COVID-19, cough and digestive disorders were more common in patients with elevated cardiac troponin on discharge, with a trend towards greater respiratory manifestations such as intermittent fever and chills, as well as new onset renal failure. No relevant differences were found in the incidence of reduced function in the pulmonary diffusion test, nausea/vomiting, hair loss, joint pain, myalgias, sweating episodes, significant weight loss, cutaneous involvement, new-onset diabetes mellitus, red eye symptoms, flushing, sleep apnea, or incident neoplasia.

## 4. Discussion

In this study, we evaluate the impact of elevated cardiac troponin levels at discharge on the prognosis following hospitalization in patients with COVID-19, and its correlation with long-term COVID-19 symptoms, using a prospective multi-center registry.

The principal results are as follows: After propensity score matching, patients discharged alive with hospitalization and COVID-19 with elevated cardiac troponin levels (1) suffer significantly higher all-cause mortality during follow-up, even when matched for previous heart disease, (2) have a more complex in-hospital course, with a higher incidence of sepsis and heart failure, and (3) are more likely to exhibit long-term COVID-19 cardiovascular traits (Central Illustration). These results are reproducible both in the matched 1:3 cohort, as well as in the overall population of our study.

It is well-established that patients with cardiovascular risk factors, particularly those with hypertension, diabetes, or underlying cardiovascular disease have worse outcomes [15,18]. In such cases, the age-adjusted Charlson comorbidity index score has been shown to be a great predictor for severe clinical outcomes in hospitalized patients with COVID-19 infection, particularly in the elderly [19], even among other classical comorbidities such as thrombocytopenia, low body mass index, anemia, and male sex in the cohort by Kim et al. [19]. Other parameters such as the neutrophil-to-lymphocyte ratio have been proven to be useful, as well as to predict in-hospital mortality during the omicron dominant period [20]. Additionally, undertreatment with angiotensin-converting enzyme inhibitors/ angiotensin II receptor blockers was listed as a main prognostic indicator of in-hospital mortality in the same cohort, underscoring the importance of this finding that might help clinicians to reduce patient mortality [20].

Moreover, some authors suggest that mortality of COVID-19 is even higher for patients with previous cardiovascular disease than in patients with previous respiratory disease [15,21]. Nevertheless, in our study, mortality remained higher in the elevated troponin cohort even after balancing previous heart disease between groups in propensity score matching. This suggests a higher mortality rate in myocardial injury associated with COVID-19 hospitalization, even in the absence of previous heart disease. Furthermore, the elevated troponin cohort had a more abrupt in-hospital clinical course with more sepsis, which is a known factor of elevated troponin and has been linked with worse cardiovascular outcomes and all-cause death [4,22]. Moreover, hypoxia-induced myocardial injury, which can occur in the setting of the aforementioned complications (i.e., sepsis) can decrease the energetic cell supply, leading to intracellular acidosis and favoring apoptosis through various mechanisms [15]. Even though inflammation has been described as one of the main underlying mechanisms regarding endothelial activation, further investigation is required to clarify this aspect, since no relationship was identified between the expression of vascular adhesion molecule-1 (VCAM-1) and intercellular adhesion molecule-1 (ICAM-1) in heart tissue regarding cytokine-mediated activation [23].

Núñez-Gil et al. [15] have previously demonstrated higher mortality in discharged patients with underlying heart disease after a COVID-19 hospitalization, particularly patients with heart failure/ cardiomyopathy, which might suggest that structurally vulnerable hearts are prone to higher frailty. On top of that, abnormal coagulation parameters have been described in patients with COVID-19, which could potentially add up to direct and indirect myocardial damage through ischemic or thrombotic events. Even though the number of events is small in our cohort, the number of relevant bleedings was higher in the elevated troponin cohort [15].

Our cohort includes a wider spectrum of patients, demonstrating that elevated troponin levels at discharge, whether in the presence of underlying heart disease or not, are associated with significantly higher all-cause mortality and readmissions during follow-up.

Long-term COVID-19 symptoms have been described, even in patients without prior cardiac disease, which might suggest relevant cardiovascular tropism of SARS-COV2 [14,23].

Potential factors associated with the pathophysiology of long-term COVID-19 symptoms might encompass: (1) damage to the cell and titular structures crucial for vascular circulation, leading to increased blood clotting; (2) the prolonged presence of viral remnants or its constituent parts in various tissues; and (3) modifications in the immune system, among additional contributors [14,23].

In a recent meta-analysis conducted by Lopez-Leon et al. [16], comprising 15 manuscripts and 47,910 patients, over 50 long-term COVID-19 symptoms were identified. According to their findings, the top five prevalent manifestations were fatigue (58%), headache (44%), attention disorder (27%), hair loss (25%), and dyspnea (24%). Similarly, fatigue emerged as the most common symptom among our patients with positive troponin levels on discharge (57.7%) and in the control group (40.6%), with dyspnea being the second most common in the whole matched cohort (39.4%), which is also in line with the previous works of Núñez-Gil et al. [14] in which patients with heart disease were more likely to exhibit long-term COVID-19 symptoms on discharge. Our study suggests that dizziness, an often-unreported symptom of long-term COVID-19, is the third most common long-term COVID-19 symptom in the positive troponin cohort. As previously described, new symptoms can arise after the COVID-19 illness or even continue after it [24]. Physicians should be aware of these symptoms, in order to properly assess their onset and minimize the risk of chronic consequences of long-term COVID-19 symptoms, and help reestablish pre-COVID-19 health in affected individuals [16].

Long-term COVID-19 symptoms resemble those of chronic fatigue syndrome (CFS), which has an unclear etiology [25,26] and has also been linked to other viruses (e.g., Cytomegalovirus and Epstein–Barr virus, among others) [16]. In their recent work, Corbalán et al. [25] propose a new parameter, the functional limitation index (FLI), which can provide an accurate diagnosis of this condition, regardless of gender, in a one-day assessment. In their work, physiological parameters of ergospirometry, such as VO2 peak and VO2 max/ VO2 ratio, are altered in patients with CFS and might partially set the biological basis for fatigue in CFS. Since there is an overlap between long-term COVID-19 symptoms and CFS, the former patients might also benefit from objective physiological tests such as ergospirometry to further characterize altered parameters in this disease, secure its diagnosis, and, eventually, lead to the development of novel therapeutic strategies [27].

## 5. Limitations

The primary limitation arises from the design, and the study’s observational nature, which could lead to selection bias among other potentially relevant biases. Additionally, variability in definitions [14,24], the nature and severity of heart disease, and index event reports might differ across institutions, countries, and even in the dominant viral strain during the exact moment of the pandemic the data were collected. The term “heart disease” is broad and encompasses a wide range of conditions, thus warranting careful interpretation. Furthermore, subjective factors, like certain symptoms and their resolution being inconsistently reported, should be interpreted with caution. The quantitative value of troponin was not used. Instead, a dichotomic classification according to the 99th percentile cut-off was used [9]. We believe that this might help to overcome the heterogeneity in a multicenter study in which different units, troponin values, and assays are employed. Nevertheless, the results should be interpreted carefully when comparing this cohort to others in which a quantitative classification of troponin was employed.

Of note, smoking status was still more prevalent in the positive troponin group after propensity score adjustment. However, when assessing the smoking status and mortality in the matched cohort, a non-significant *p*-value was obtained. Nevertheless, this fact, together with the previous limitations, should be carefully considered.

The Delphi consensus, elaborated by the WHO, has offered an official definition of the post-COVID-19 condition [24]. According to the WHO, this condition typically occurs in individuals with a probable or confirmed SARS-CoV-2 infection, manifesting symptoms three months after the initial infection that can persist for at least two months and cannot be attributed to another plausible diagnosis. However, our research did not entirely align with these criteria, as its design was established prior to the release of this consensus [14,24]. Therefore, the expression “long-term COVID-19 symptoms” was used throughout the text.

According to the LONG-COVID-EXP-CM Multicenter Study [22], a more critical presentation of long-term COVID-19 can be linked to more developed symptoms. Our study does not capture the evolution time of the symptoms, so this trait could not be assessed [28]. Multi-specialty management in long-term COVID-19 symptom clinics could enhance diagnosis and recovery [29].

## 6. Conclusions

Our findings underscore the importance of long-term COVID-19 symptoms in clinical practice, particularly among patients with elevated troponin levels at discharge, as they face higher mortality rates and are more prone to experiencing cardiovascular traits of long-term COVID-19. Physicians should be equipped to promptly identify these patients and consider closer follow-up.

## Figures and Tables

**Table 1:** Baseline characteristics of the general population discharged alive from the hospital after propensity score matching. Data were calculated over the available population with the information unless indicated otherwise with a fraction sign “/” using the available subjects for such parameter. Follow-up time is expressed as days (median) and interquartile range (IQR). Parameters included in the propensity score matching analysis are outlined in *italics*. *p*-Values marked with an asterisk (*) were corrected using a Fisher’s exact test.

		Matched Population (n = 312)	Elevated Troponin on Discharge (n = 78)	Not Elevated Troponin on Discharge (n = 234)	*p*-Value
*Age (years)*		65.76 ± 14.90	65.69 ± 14.49	65.78 ± 14.73	0.966
*Male*		188 (60.3%)	46 (59%)	142 (60.7%)	0.789
*Hypertension*		196 (62.8%)	50 (64.1%)	146 (62.4%)	0.787
*Obesity*		60 (19.2%)	14 (17.9%)	46 (19.7%)	0.740
*Diabetes Mellitus*		63 (20.2%)	17 (21.8%)	46 (19.7%)	0.684
Dislipidaemia		107 (34.3%)	27 (34.6%)	80 (34.2%)	0.945
Active smoking		42 (14%)	19 (25.7%)	23 (10.2%)	0.001
Renal failure		21 (6.7%)	8 (10.3%)	13 (5.6%)	0.151
Lung disease		68 (23.7%)	14 (20.3%)	54 (24.8%)	0.446
*Heart disease*		130 (41.67%)	32 (41%)	98 (41.9%)	0.895
Cerebrovascular disease		22 (7.1%)	6 (7.7%)	16 (6.8%)	0.798
Connectivopathy		8 (2.6%)	1 (1.3%)	7 (3%)	0.684
Liver disease		7 (2.2%)	2 (2.6%)	5 (2.1%)	1.000
Cancer status		34 (10.9%)	8 (10.3%)	26 (11.1%)	0.834
Inmunosupression		21 (6.7%)	6 (7.7%)	15 (6.4%)	0.696
In hospital complications	Respiratory insufficiency	135 (43.5%)	38 (48.7%)	97 (41.5%)	0.262
	Heart failure	29 (9.3%)	16 (20.5%)	13 (5.6%)	<0.001
	Renal failure	41 (13.1%)	15 (19.2%)	26 (11.1%)	0.066
	Upper respiratory tract infection	39 (12.5%)	11 (14.1%)	28 (12%)	0.621
	Pneumonia	231 (76.2%)	60 (76.9%)	171 (76%)	0.869
	Sepsis	36 (11.5%)	26 (33.3%)	10 (4.3%)	<0.001
	Systemic inflammatory response syndrome	36 (11.5%)	7 (9%)	29 (12.4%)	0.413
	Relevant bleeding	11 (3.5%)	6 (7.7%)	5 (2.1%)	0.032
	Hemoptysis	9 (2.9%)	6 (7.7%)	3 (1.3%)	0.009 *
	Embolic events	6 (1.9%)	3 (3.8%)	3 (1.3%)	0.168
Hospital readmission for any cause		81 (26%)	37 (47.4%)	44 (18.8%)	<0.001
All-cause death		19 (6.1%)	14 (17.9%)	5 (2.1%)	<0.001
Follow-up		12.97; 8.60–14.79	12.60; 7.07–13.23	13; 9.60–15.54	-

**Table 2:** Long-term COVID-19 symptoms according to discharge troponin status after propensity score matching. Data were calculated over the available population with the information unless indicated otherwise with a fraction sign “/” using the available subjects for such parameter. *p*-values marked with an asterisk (*) were corrected using a Fisher’s exact test.

		Matched Population (n = 312)	Elevated Troponin on Discharge (n = 78)	Not Elevated Troponin on Discharge (n = 234)	*p*-Value
Any long-term COVID-19 symptoms		210 (67.3%)	57 (73.1%)	153 (65.4%)	0.210
Long-term COVID-19 cardiovascular traits	Fatigue	140 (44.9%)	45 (57.7%)	95 (40.6%)	0.009
	Dyspnea	123 (39.4%)	49 (62.8%)	74 (31.6%)	<0.001
	Dizziness	48 (15.4%)	23 (29.5%)	25 (10.7%)	<0.001
	Chest pain	36 (11.5%)	15 (19.2%)	21 (9%)	0.014
	Acute coronary syndrome	9 (2.9%)	6 (7.7%)	3 (1.3%)	0.009 *
	Palpitations	47 (15.1%)	23 (29.5%)	24 (10.3%)	<0.001
	Increased resting heart rate	13 (10.6%)	14 (17.9%)	19 (8.1%)	0.015
	Syncope	6 (1.9%)	2 (2.6%)	4 (1.7%)	0.642 *
	Arrhythmia	41 (13.1%)	20 (25.6%)	21 (9%)	<0.001
	Atrial fibrillation	32 (10.3%)	10 (12.8%)	22 (9.4%)	0.389
	Peri/myocarditis	5 (1.6%)	5 (6.4%)	0 (0%)	0.001 *
	Inferior limb edema	24 (7.7%)	12 (15.4%)	12 (5.1%)	0.003
	New onset hypertension	13 (4.2%)	3 (3.8%)	10 (4.3%)	1.000
	New onset ventricular dysfunction	21 (6.7%)	15 (19.2%)	6 (2.6%)	<0.001
	Relevant bleeding	2 (0.6%)	1 (1.3%)	1 (0.4%)	0.438 *
Long-term COVID-19 neuro-psychological traits	Headache	27 (8.7%)	3 (3.8%)	24 (10.3%)	0.081 *
	Migraine	13 (4.2%)	1 (1.3%)	12 (5.1%)	0.197 *
	Ageusia	22 (7.1%)	5 (6.4%)	17 (7.3%)	0.798
	Anosmia	19 (6.1%)	7 (9%)	12 (5.1%)	0.272
	Attention disorder	26 (8.3%)	9 (11.5%)	7 (7.3%)	0.237
	Memory loss	11 (9.9%)	10 (12.8%)	21 (9%)	0.325
	Cognitive impairment	21 (6.7%)	8 (10.3%)	13 (5.6%)	0.151
	Anxiety	50 (16%)	17 (21.8%)	33 (14.1%)	0.109
	Depression	33 (10.6%)	13 (16.7%)	20 (8.5%)	0.043
	Tinnitus or hearing loss	13 (4.2%)	6 (7.7%)	7 (3%)	0.098 *
	Sleep disorder	46 (14.7%)	10 (12.8%)	36 (15.4%)	0.580
	Mood disorder	39 (12.5%)	14 (17.9%)	25 (10.7%)	0.093
	Paraonia	10 (3.2%)	5 (6.4%)	5 (2.1%)	0.128
Other long-term COVID-19 symptoms	Cough	47 (15.1%)	21 (26.9%)	26 (11.1%)	0.001
	Reduced pulmonary diffusion test	31 (9.9%)	13 (16.7%)	18 (7.7%)	0.022
	Polypnea	18 (5.8%)	8 (10.3%)	10 (4.3%)	0.087
	Sleep apnea	15 (4.8%)	5 (6.4%)	10 (4.3%)	0.540
	Digestive disorders	22 (7.1%)	10 (12.8%)	12 (5.1%)	0.022
	Nausea/Vomiting	13 (4.2%)	5 (6.4%)	8 (3.4%)	0.323
	Intermittent fever	17 (5.4%)	8 (10.3%)	9 (3.8%)	0.042
	Chills	17 (5.4%)	9 (11.5%)	8 (3.4%)	0.017
	Hair loss	20 (6.4%)	5 (6.4%)	15 (6.4%)	1.000
	Joint pain	34 (10.9%)	7 (9%)	27 (11.5%)	0.529
	Myalgias	34 (10.9%)	5 (6.4%)	29 (12.4%)	0.142
	Significant sweating episodes	8 (2.6%)	1 (1.3%)	7 (3%)	0.684 *
	Significant weight loss	24 (7.7%)	3 (3.8%)	21 (9%)	0.141 *
	Cutaneous involvement	14 (4.5%)	3 (3.8%)	11 (4.7%)	1.000 *
	New onset diabetes mellitus	7 (2.2%)	2 (2.6%)	5 (2.1%)	1.000 *
	New onset renal failure	22 (7.1%)	13 (16.7%)	9 (3.8%)	<0.001
	Red eye symptoms	4 (1.3%)	0 (0%)	4 (1.7%)	0.575 *
	Flushing	1 (0.3%)	0 (0%)	1 (0.4%)	1.000 *
	Incident neoplasia	6 (1.9%)	3 (3.8%)	3 (1.3%)	0.168

## Data Availability

Data is available upon reasonable request to the corresponding author.

## Supplementary Materials

The following supporting information can be downloaded at: https://www.mdpi.com/article/10.3390/jcm13092596/s1, Figure S1: Survival analysis represented by Kaplan-Meier curves; Table S1: Baseline values before and after propensity score matching; Table S2: Baseline characteristics of the general population discharged alive from the hospital after propensity score matching; Table S3: Long-term COVID-19 symptoms according to discharge troponin status after propensity score matching.
