# Supplementary material for: Elevated Troponins after COVID-19 Hospitalization and Long-Term COVID-19 Symptoms: Incidence, Prognosis, and Clinical Outcomes—Results from a Multi-Center International Prospective Registry (HOPE-2)
Source: J Clin Med. 2024 Apr 28;13(9):2596. doi: 10.3390/jcm13092596 (PMC11084489; doi:10.3390/jcm13092596)
Supplement: Supplementary file 1 [file jcm-13-02596-s001.zip › jcm-2932682-supplementary.pdf]

Journal of
Clinical Medicine

MDPI

Supplementary Table S1. Baseline variables introduced in the univariant model are located in the rows, the selected variables for propensity score matching are outlined in bold and italics; with their p value before and after the matching attached.

	Baseline values			After 1:3 propensity score matching		
	**Elevated troponin on discharge (n=78)**	**Not elevated troponin on discharge (n=2304)**	***p* value**	**Elevated troponin on discharge (n=78)**	**Not elevated troponin on discharge (n=234)**	***p* value**
**Age (years)**	65.69±15.49	59.14±17.44	***0.001***	65.69±15.49	65.78±14.73	0.966
Gender (male)	46 (58.9%)	1322 (57.4%)	0.779	46 (59%)	142 (60.7%)	0.789
**Hypertension**	50 (64.1%)	990 (42.9%)	***<0.001***	50 (64.1%)	146 (64.2%)	0.787
Obesity	14 (17.9%)	339 (16.9%)	0.813	14 (17.9%)	46 (19.7%)	0.740
**Diabetes mellitus**	17 (21.8%)	339 (14.7%)	***0.084***	17 (21.8%)	46 (19.7%)	0.684
**Previous heart disease**	32 (41%)	381 (16.5%)	***<0.001***	32 (41%)	98 (41.9%)	0.895

Supplementary Table S2. Baseline characteristics of the general population discharged alive from the hospital after propensity score matching. Data has been calculated over the available population with the needed information, unless indicated otherwise with a fraction sign "/" using the available subjects for such parameter. Follow-up time is expressed as days (median) and interquartile range (IQR). Parameters included in the propensity score matching analysis are outlined in *italics*. P-values marked with an asterisk (*) were corrected using the Fisher´s exact test.

		Population (n=2382)	Elevated troponin on discharge (n=78)	Not elevated troponin on discharge (n=2304)	p value
Age (years)		59.3±17.4	65.69±15.49	59.1±17.4	0.001
Male		1368 (57.4%)	46 (59%)	1322 (57.4%)	0.779
Hypertension		1040 (43.7%)	50 (64.1%)	990 (43%)	<0.001
Obesity		404 (17%)	14 (17.9%)	390 (16.9%)	0.813
Diabetes Mellitus		356 (14.9%)	17 (21.8%)	339 (14.7%)	0.084
Dislipidaemia		723 (30.4%)	27 (34.6%)	696 (30.2%)	0.405
Active smoking		232 (9.7%)	19 (25.7%)	213 (9.5%)	<0.001
Renal failure		95 (4%)	8 (10.3%)	87 (3.8%)	0.004
Lung disease		353 (14.8%)	14 (20.3%)	339 (17.6%)	0.567
Heart disease		413 (17.3%)	32 (41%)	381 (16.5%)	<0.001
Cerebrovascular disease		124 (5.2%)	6 (7.7%)	118 (5.1%)	0.315
Connectivopathy		60 (2.5%)	1 (1.3%)	59 (2.6%)	0.436*
Liver disease		63 (2.6%)	2 (2.6%)	61 (2.6%)	0.964*
Cancer status		256 (10.7%)	8 (10.3%)	248 (10.8%)	0.887
Inmunosupression		150 (6.3%)	6 (7.7%)	144 (6.3%)	0.606
In hospital complications	Respiratory insufficiency	946 (39.7%)	38 (48.7%)	908 (39.4%)	0.098
	Heart failure	106 (4.5%)	16 (20.5%)	90 (3.9%)	<0.001
	Renal failure	194 (8.1%)	15 (19.2%)	179 (7.8%)	<0.001
	Upper respiratory tract infection	254 (10.7%)	11 (14.1%)	243 (10.5%)	0.317
	Pneumonia	1718 (73.9%)	60 (76.9%)	1658 (73.8%)	0.531
	Sepsis	159 (6.7%)	26 (33.3%)	133 (5.8%)	<0.001

	Systemic inflammatory response syndrome	256 (10.7%)	7 (9%)	249 (10.8%)	0.607
	Relevant bleeding	52 (2.2%)	6 (7.7%)	46 (2%)	0.001
	Hemoptysis	35 (1.5%)	6 (7.7%)	29 (1.3%)	<0.001
	Embolic events	39 (1.6%)	3 (3.8%)	36 (1.6%)	0.180*
Hospital readmission for any cause		388 (16.3%)	37 (47.4%)	351 (15.2%)	<0.001
All-cause death		126 (5.3%)	14 (17.9%)	112 (4.9%)	<0.001
Follow-up		12.08;1.13-13.23	12.60;7.07-13.23	12.03;0.94-13.23	-

Supplementary Table S3 Long-term COVID-19 symptoms according to discharge troponin status after propensity score matching. Data has been calculated over the available population with the information, unless indicated otherwise with a fraction sign "/" using the available subjects for such parameter. P-values marked with an asterisk (*) were corrected using the Fisher´s exact test.

		Population (n=2382)	Elevated troponin on discharge (n=78)	Not elevated troponin on discharge (n=2304)	p value
Any long-term COVID-19 symptoms		1307 (54.9%)	59 (75.6%)	1248 (54.2%)	<0.001
Long-term COVID-19 Cardiovascular traits	Fatigue	692 (29.1%)	45 (57.7%)	647 (28.1%)	<0.001
	Dyspnea	585 (24.6%)	49 (62.8%)	536 (62.8%)	<0.001
	Dizziness	182 (7.6%)	23 (29.5%)	159 (6.9%)	<0.001
	Chest pain	191 (8%)	15 (19.2%)	176 (7.6%)	<0.001
	Acute coronary syndrome	27 (1.1%)	6 (7.7%)	21 (0.9%)	<0.001
	Palpitations	221 (9.3%)	23 (29.5%)	198 (8.6%)	<0.001
	Increased resting heart rate	119 (5%)	14 (17.9%)	105 (4.6%)	<0.001
	Syncope	28 (1.2%)	2 (2.6%)	26 (1.1%)	0.247*
	Arrhythmia	141 (5.9%)	20 (25.6%)	121 (5.3%)	<0.001
	Atrial fibrillation	104 (4.4%)	10 (12.8%)	94 (4.1%)	<0.001
	Peri/myocarditis	12 (0.5%)	5 (6.4%)	7 (0.3%)	<0.001
	Inferior limb edema	84 (3.5%)	12 (15.4%)	72 (3.1%)	<0.001
	New onset hypertension	59 (2.5%)	3 (3.8%)	56 (2.4%)	0.462*
	New onset ventricular dysfunction	43 (1.8%)	15 (19.2%)	28 (1.2%)	<0.001
	Relevant bleeding	21 (0.9%)	1 (1.3%)	20 (0.9%)	0.718*
Long-term COVID-19 neuro-psychological traits	Headache	144 (6%)	3 (3.8%)	141 (6.1%)	0.377*
	Migraine	64 (2.7%)	1 (1.3%)	63 (2.7%)	0.387*
	Ageusia	119 (5%)	5 (6.4%)	114 (4.9%)	0.560
	Anosmia	102 (4.3%)	7 (9%)	95 (4.1%)	0.037
	Attention disorder	114 (4.8%)	9 (11.5%)	105 (4.6%)	0.005
	Memory loss	156 (6.5%)	10 (12.8%)	146 (6.3%)	0.023
	Cognitive impairment	93 (3.9%)	8 (10.3%)	85 (3.7%)	0.003
	Anxiety	274 (11.5%)	17 (21.8%)	257 (11.2%)	0.004
	Depression	183 (7.7%)	13 (16.7%)	170 (7.4%)	0.002
	Tinnitus or hearing loss	59 (2.5%)	6 (7.7%)	53 (2.3%)	0.003
	Sleep disorder	196 (8.2%)	10 (12.8%)	186 (8.1%)	0.133

	Mood disorder	143 (6%)	14 (17.9%)	129 (5.6%)	<0.001
	Paraonia	44 (1.8%)	5 (6.4%)	39 (1.7%)	0.002
Other long-term COVID-19 symptoms	Cough	261 (11%)	21 (26.9%)	240 (10.4%)	<0.001
	Reduced pulmonary diffusion test	178 (7.5%)	13 (16.7%)	165 (7.2%)	0.002
	Polypnea	69 (2.9%)	8 (10.3%)	61 (2.6%)	<0.001
	Sleep apnea	41 (1.7%)	5 (6.4%)	36 (1.6%)	0.001
	Digestive disorders	124 (5.2%)	10 (12.8%)	114 (4.9%)	0.002
	Nausea/Vomiting	54 (2.3%)	5 (6.4%)	49 (2.1%)	0.012
	Intermittent fever	55 (2.3%)	8 (10.3%)	47 (2%)	<0.001
	Chills	46 (1.9%)	9 (11.5%)	37 (1.6%)	<0.001
	Hair loss	117 (4.9%)	5 (6.4%)	112 (4.9%)	0.534
	Joint pain	156 (6.5%)	7 (9%)	149 (6.5%)	0.379
	Myalgias	185 (7.8%)	5 (6.4%)	180 (7.8%)	0.649
	Significant sweating episodes	27 (1.1%)	1 (1.3%)	26 (1.1%)	0.902*
	Significant weight loss	130 (5.5%)	3 (3.8%)	127 (5.5%)	0.503*
	Cutaneous involvement	67 (2.8%)	3 (3.8%)	64 (2.8%)	0.594*
	New onset diabetes mellitus	31 (1.3%)	2 (2.6%)	29 (1.3%)	0.375*
	New onset renal failure	67 (2.8%)	13 (16.7%)	54 (2.3%)	<0.001
	Red eye symptoms	29 (1.2%)	0 (0%)	29 (1.3%)	0.163*
	Flushing	10 (0.4%)	0 (0%)	10 (0.4%)	0.414*
	Incident neoplasia	23 (1%)	3 (3.8%)	20 (0.9%)	0.043*

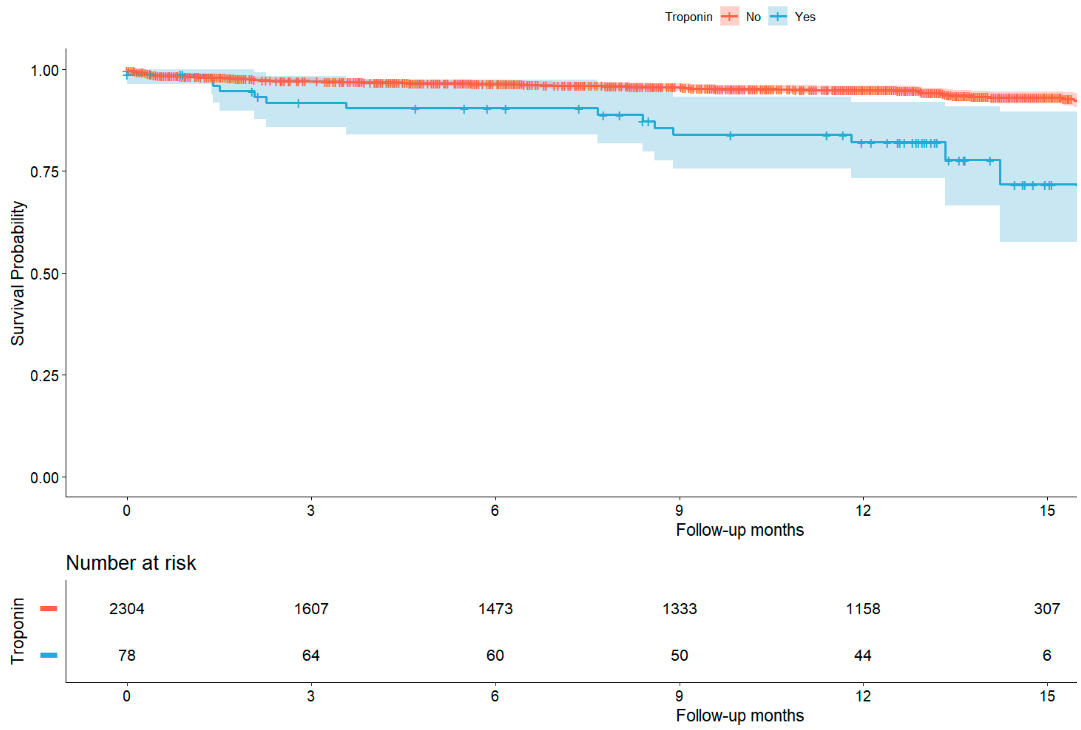

Supplementary Figure S1. Survival analysis represented by Kaplan-Meier curves (upper part) and number of patients at risk after the end of each time period (lower part). The shaded area surrounding each curve represents the 95% confidence interval; demonstrating greater all-cause mortality with a Log-Rank (Mantel-Cox) test of 27.70 (p<0.001) in patients with elevated troponin after hospital discharge in the overall population.
